# Supplementary material for: Structural basis for VPS34 kinase activation by Rab1 and Rab5 on membranes
Source: Nat Commun. 2021 Mar 10;12:1564. doi: 10.1038/s41467-021-21695-2 (PMC7946940; doi:10.1038/s41467-021-21695-2)
Supplement: Supplementary file 1 — Supplementary Information [file 41467_2021_21695_MOESM1_ESM.docx]

**Supplementary Information**

Structural basis for VPS34 kinase activation by Rab1 and Rab5 on membranes

**Authors:** Authors: Shirley Tremel^1^, Yohei Ohashi^1^, Dustin R. Morado^1,2^, Jessie Bertram^1^, Olga Perisic^1^, Laura T. L. Brandt^1^, Marie-Kristin von Wrisberg^3^, Zhuo A. Chen^4^, Sarah L. Maslen^1^, Oleksiy Kovtun^1^, Mark Skehel^1^, Juri Rappsilber^4,5^, Kathrin Lang^3^, Sean Munro^1^*, John A. G. Briggs^1^*, Roger L. Williams^1^*

^1^MRC Laboratory of Molecular Biology, Cambridge, UK

^2^Science for Life Laboratory, Department of Biochemistry and Biophysics, Stockholm University, Solna, Sweden

^3^Center for Integrated Protein Science Munich (CIPSM), Department of Chemistry, Lab for Synthetic Biochemistry, Technical University of Munich, Institute for Advanced Study, TUM-IAS, Garching, Germany

^4^Bioanalytics, Institute of Biotechnology, Technische Universität Berlin, Berlin, Germany

^5^Wellcome Centre for Cell Biology, University of Edinburgh, Edinburgh, UK

*Corresponding authors: sean@mrc-lmb.cam.ac.uk, jbriggs@mrc-lmb.cam.ac.uk, [rlw@mrc-lmb.cam.ac.uk](mailto:rlw@mrc-lmb.cam.ac.uk)

**Contents:**

**Supplementary Figs. 1-10**

**Supplementary Tables 1-4**

Supplementary Data 1 and 2 are provided separately as Excel files

Supplementary Movie1 is provided separately as a .mov file

Supplementary Movie2 is provided separately as a .mp4 file


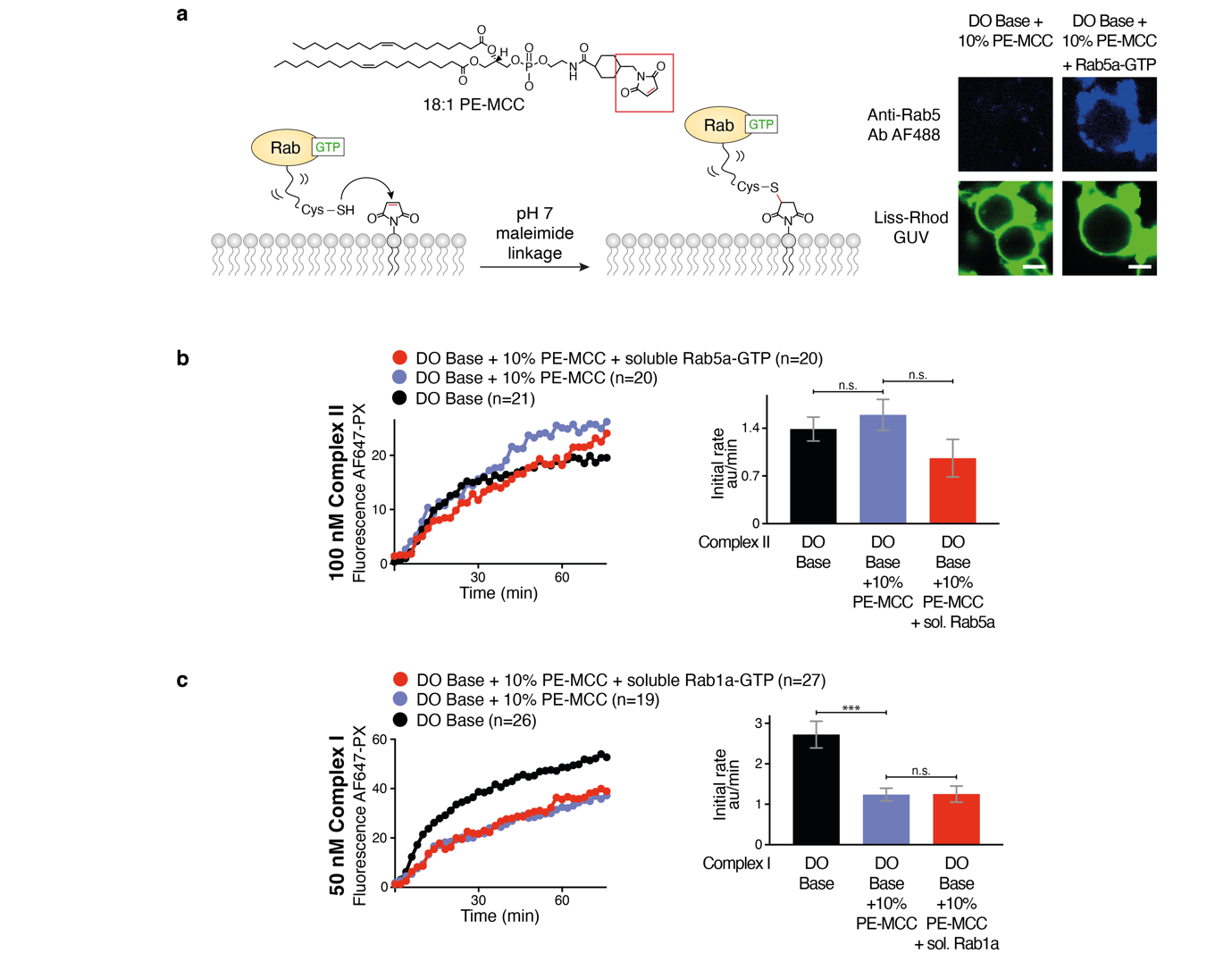


**Supplementary Figure 1 | GUV assays of VPS34 complexes by membrane-associated or soluble Rab-GTP.**

**a,** A schematic for covalent coupling of Rabs to membranes by maleimide linkage (left). Both Rab1a and Rab5a had surface-exposed Cys residues mutated (human Rab5a C19S, C63S; human Rab1a, C26S, C126S), to facilitate formation of a stable thioether bond between a C-terminal Cys residue (human Rab5a ending at Cys212; human Rab1a ending at Cys204) and the maleimide-functionalized (18:1 PE-MCC) lipid vesicles. Covalent attachment of Rab5a-GTP to GUVs containing 10% PE-MCC was visualized by immunofluorescence using an anti-Rab5 antibody and AlexaFluor 488-labeled secondary antibody (right). The GUV membranes were visualized by 0.1% Lissamine-Rhodamine-DOPE incorporated in the lipid mixture. Scale bars: 5 μm. **b,** Activity of complex II on GUVs with or without 10% PE-MCC and soluble Rab5a-GTP. Neither the presence of 10% PE-MCC in the GUV membranes nor the presence of soluble Rab5-GTP in the reaction has an effect on complex II activity. On the right, the initial rates (AF647-PX fluorescence change/min in arbitrary units, AU) are depicted. **c,** Activity of complex I on GUVs with or without 10% PE-MCC and soluble Rab1a-GTP. The presence of 10% PE-MCC in membranes decreases basal activity of complex I on GUVs. Soluble Rab1a-GTP has no effect on complex I activity on GUVs. On the right, the initial rates (AF647-PX fluorescence change/min in arbitrary units, AU) are depicted. ***: p<0.001; n.s.: p> 0.05. Source data are provided as a Source Data file.

**Supplementary Figure 2 | Vesicle flotation assays for interaction of complexes I and II with Rab5a- or Rab1-coupled vesicles.**

Rab-specific complex recruitment to 100 nm liposomes (LUVs) using lipid flotation assays. VPS34 complex I or II is mixed with LUVs coupled to Rab5a or Rab1a and the mixture was added on top of a sucrose gradient and centrifuged. Membrane-bound proteins float up to the top of the gradient as seen by gel analysis of gradient fractions. Left: gel images. Right: quantification of the left gels. **a,** Complex II with Rab5a-GTP or Rab5a-GDP (the same gel as shown in Fig. 1c). The experiment was performed three times and one representative experiment is shown. **b,** Complex I with with Rab5a-GTP or Rab5a-GDP. The experiment was performed once. **c,** Complex I with Rab1a-GTP or Rab1a-GDP (the same gel as shown in Fig. 3d). The experiment was performed once. **d,** Complex II with Rab1a-GTP. The experiment was performed once. Source data are provided as a Source Data file.


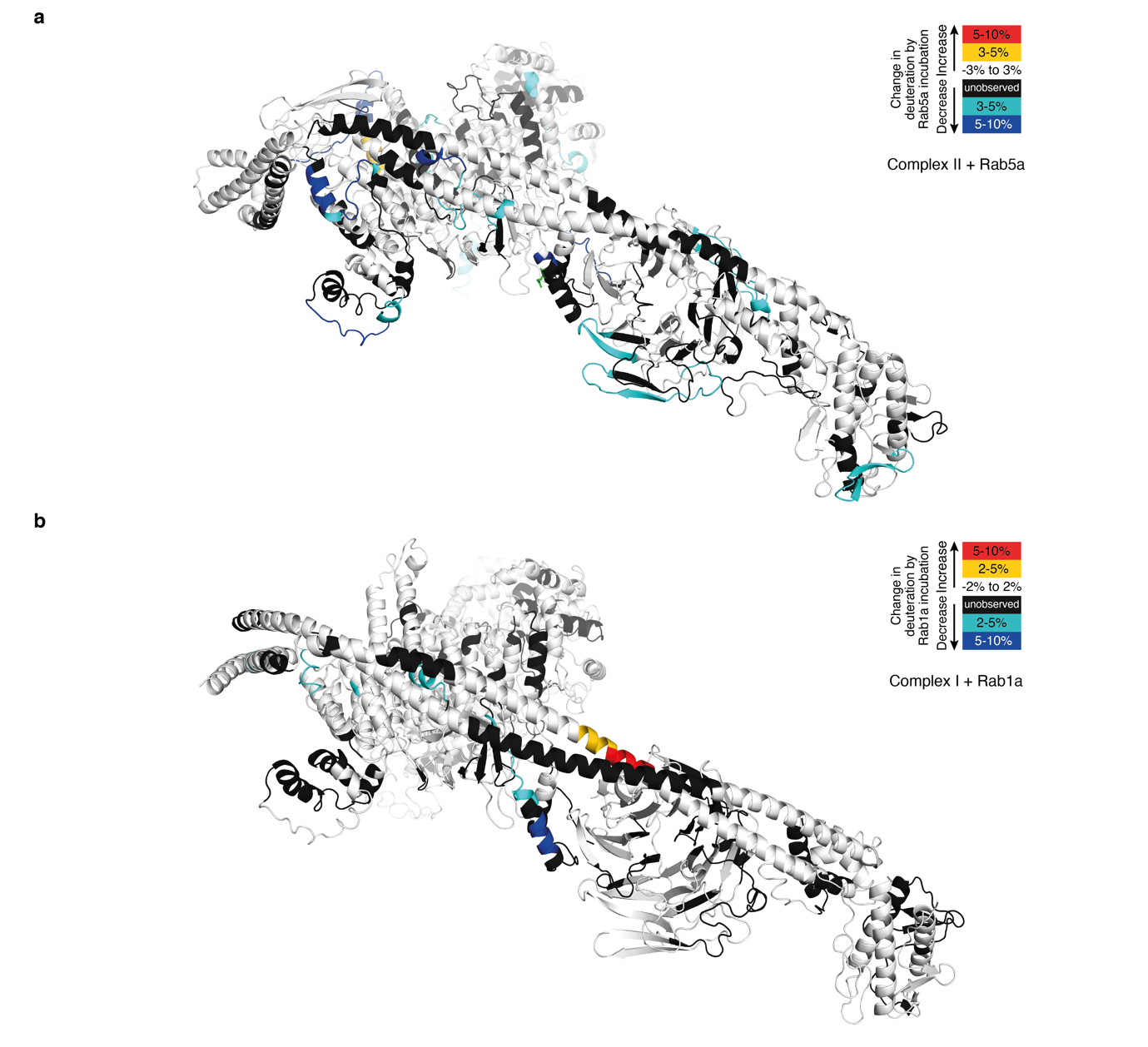


**Supplementary Figure 3**

**a,** Mapping the Rab5a binding site on complex II by the HDX-MS. HDX changes are displayed on a model of human complex II. Rab5a binding protects (colored in cyan and blue) the VPS34 C2 helical hairpin insertion (C2HH) and the VPS15 SGD and WD40 domains (as shown in Fig. 1e). Residues that are not covered by any peptide in the HDX-MS analysis are colored black. **b,** Mapping Rab1a binding site on complex I by HDX-MS. Rab1a binding increases protection of the VPS34 C2 insertion (C2HH), and decreases protection of Beclin 1 CC2 (as in Fig. 3e).

**
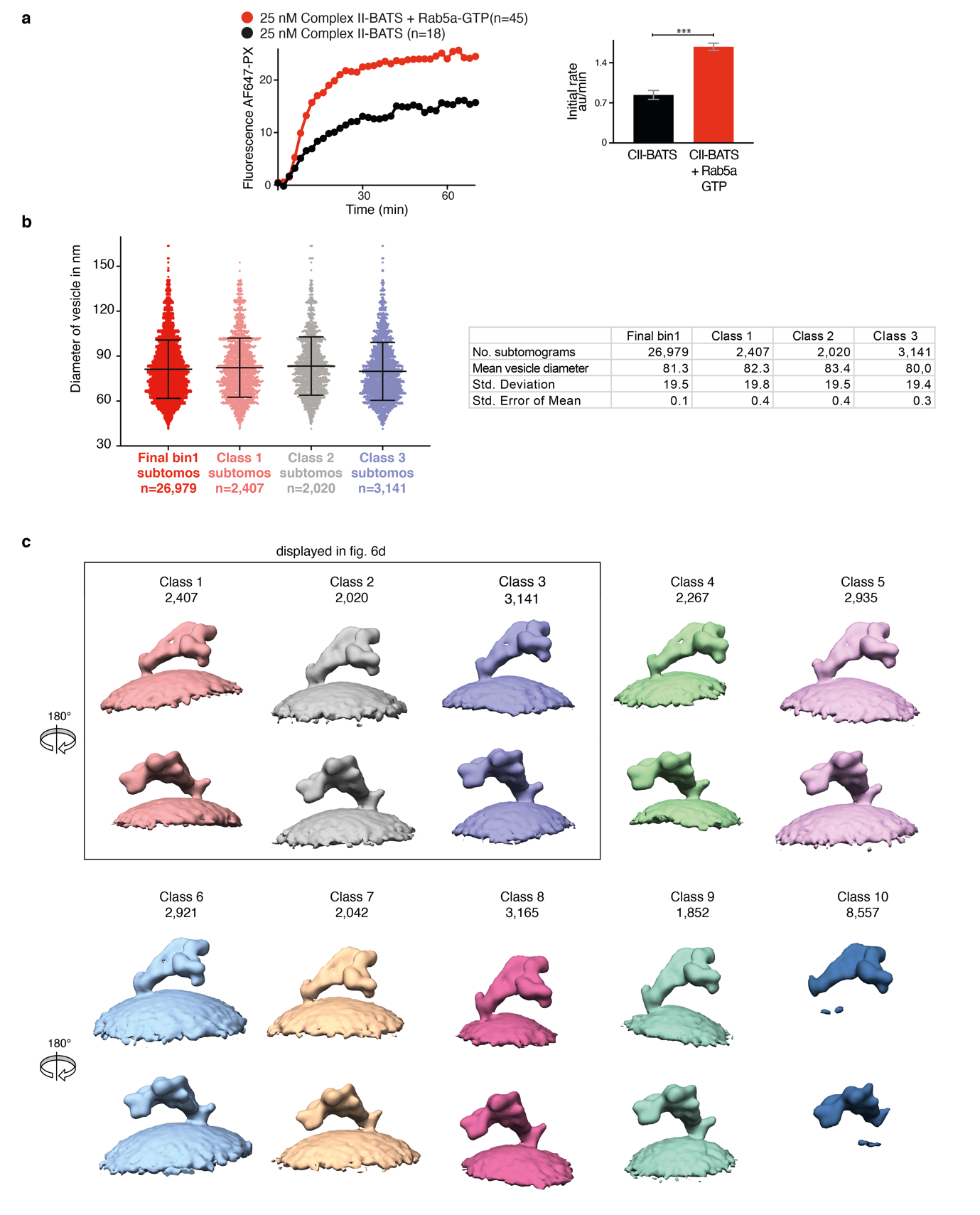
**

**Supplementary Figure 4 | Analysis of the orientation of the complex relative to the membrane by cryo-ET 3D classification.**

**a,** The activity of complex II in which the C-terminus of UVRAG was deleted (residues 1-464 remaining) and replaced by the BATS domain of ATG14L (residues 413-492). Complex II-BATS is still activated by Rab5-GTP. On the right, the initial rates (AF647-PX fluorescence change/min in arbitrary units, AU) are depicted. ***: p<0.001. **b,** The vesicle diameters were determined by calculating the vesicle centroid by the average position of the subtomograms around each vesicle after membrane alignment. Then an average diameter was calculated by measuring the distance of each subtomogram to the calculated centroid and multiplied by two. The distance between the protein complex and membrane (~6 nm) was subtracted from the calculated diameter. For each subtomogram, the corresponding average vesicle diameter is plotted (left, data are shown as the mean ± standard deviation) and the mean of the vesicle diameter distribution is shown (right). Classes 1-3 from the 3D classification have different orientations relative to the membrane (see panel c), but have similar vesicle diameter distributions, suggesting that different orientations of the complex II with respect to the membrane are not caused by different membrane curvatures. **c,** 10 classes obtained from bin4 3D classification of the membrane region are displayed. For each class, the number of particles is labelled above. Classes 1-3 were further aligned in bin2 and are displayed in Fig. 6d. Source data are provided as a Source Data file.

**
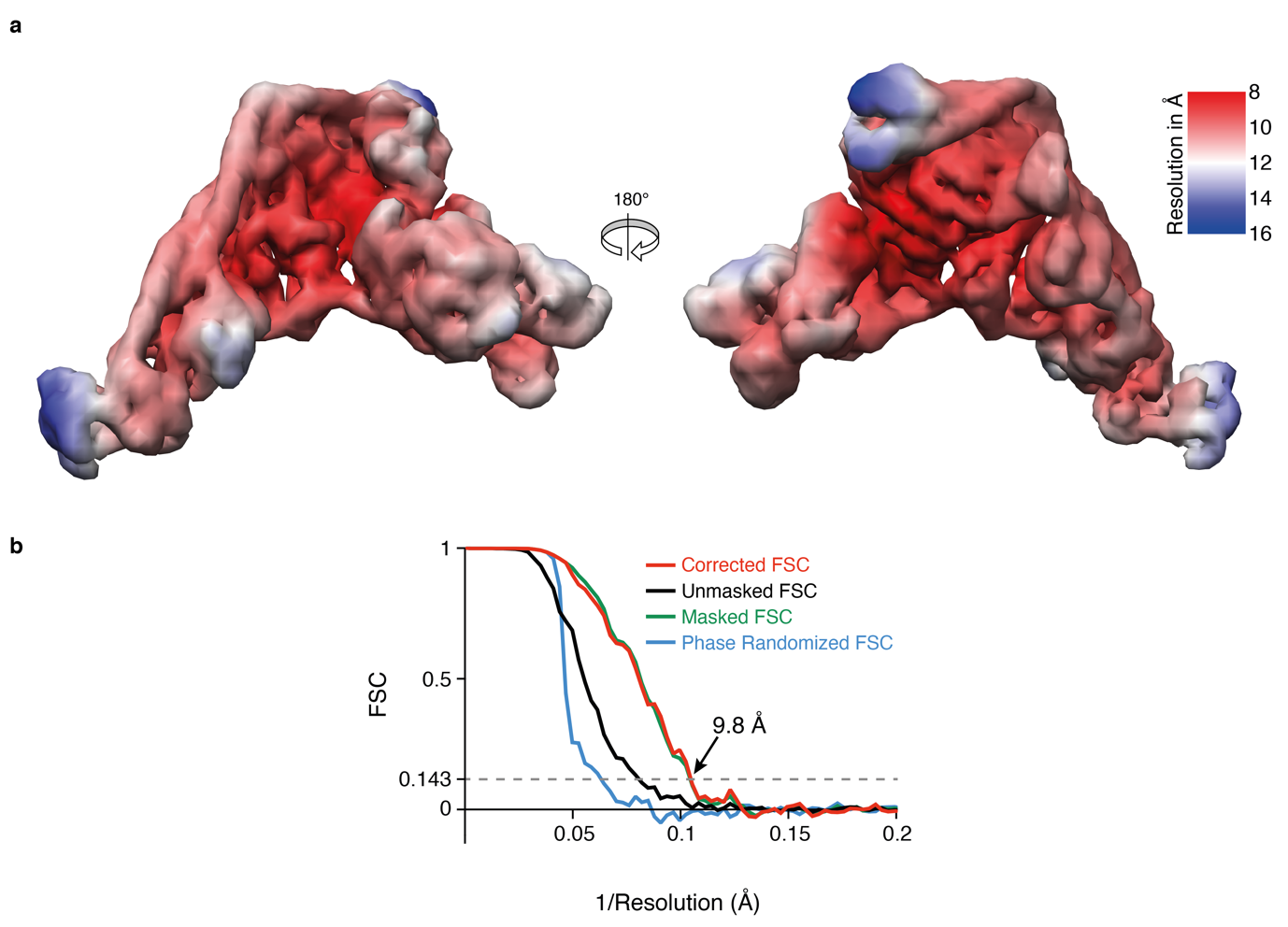
Supplementary Figure 5 | Density map for complex II determined by subtomogram averaging.**

**a,** Map after local-denoising with the LAFTER algorithm^76^ and coloured by local resolution calculated in Relion 3.0^75^ according to the indicated colour map. **b,** FSC curves for corrected, unmasked, masked and phase randomized reconstructions. The 9.8 Å overall resolution at the 0.143 criterion is marked by an arrow. Source data are provided as a Source Data file.


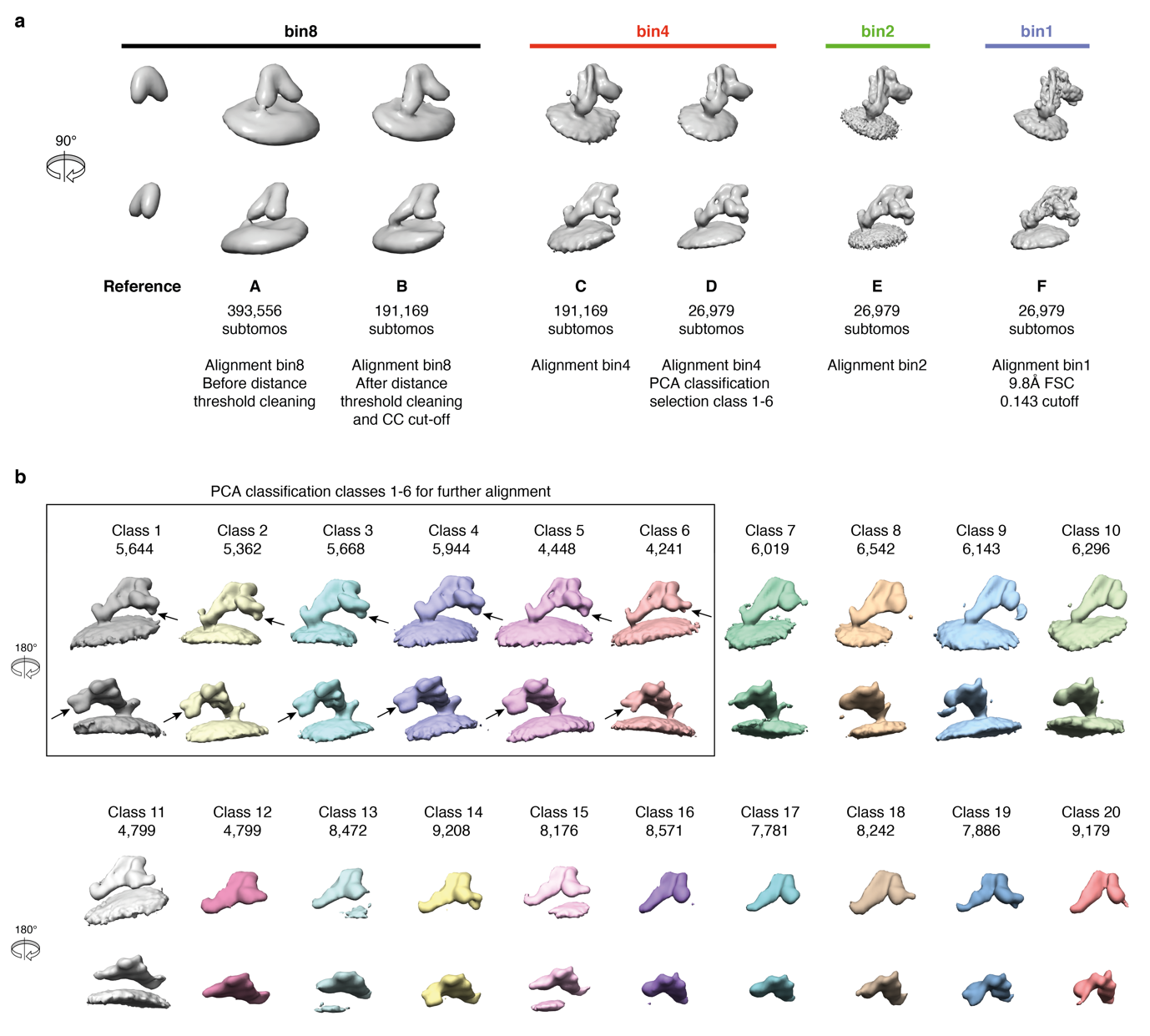


**Supplementary Figure 6 | Subtomogram averaging strategy.**

**a,** Subtomograms were aligned to a V-shaped reference until subtomograms converged and formed clusters (A). Subtomogram coordinates were cleaned by a minimal distance threshold and cross correlation cut-off so that 191,169 subtomograms remained (B). Subtomograms were split into even/odd halves in bin4 and further aligned separately until resolution converged (C). In order to analyse the heterogeneity of the subtomograms, a principal component analysis (PCA) classification on wedge-masked difference maps was used to classify the protein complex region of the subtomograms^52^. The data was sorted into 20 classes (**b**) and classes 1-6 were combined to a total of 26,979 subtomograms and further aligned in bin4 (D). The alignment was then continued in bin2, where the subtomograms were shifted to the centre of the box (E), and in bin1 until resolution converged (F). The local resolution calculated in Relion 3.0^75^ showed a range of 8-16 Å with an overall resolution 9.8 Å (FSC 0.143 cutoff) (Supplementary Figure 5). **b,** Analysis of subtomogram heterogeneity by 3D classification. 20 classes obtained from bin4 3D classification of the protein complex region in the subtomograms. For each class, the number of particles is labelled above. Classes 1-6, with the most distinct features, were chosen for further alignment. Arrows: VPS34/VPS15 kinase domains.

**
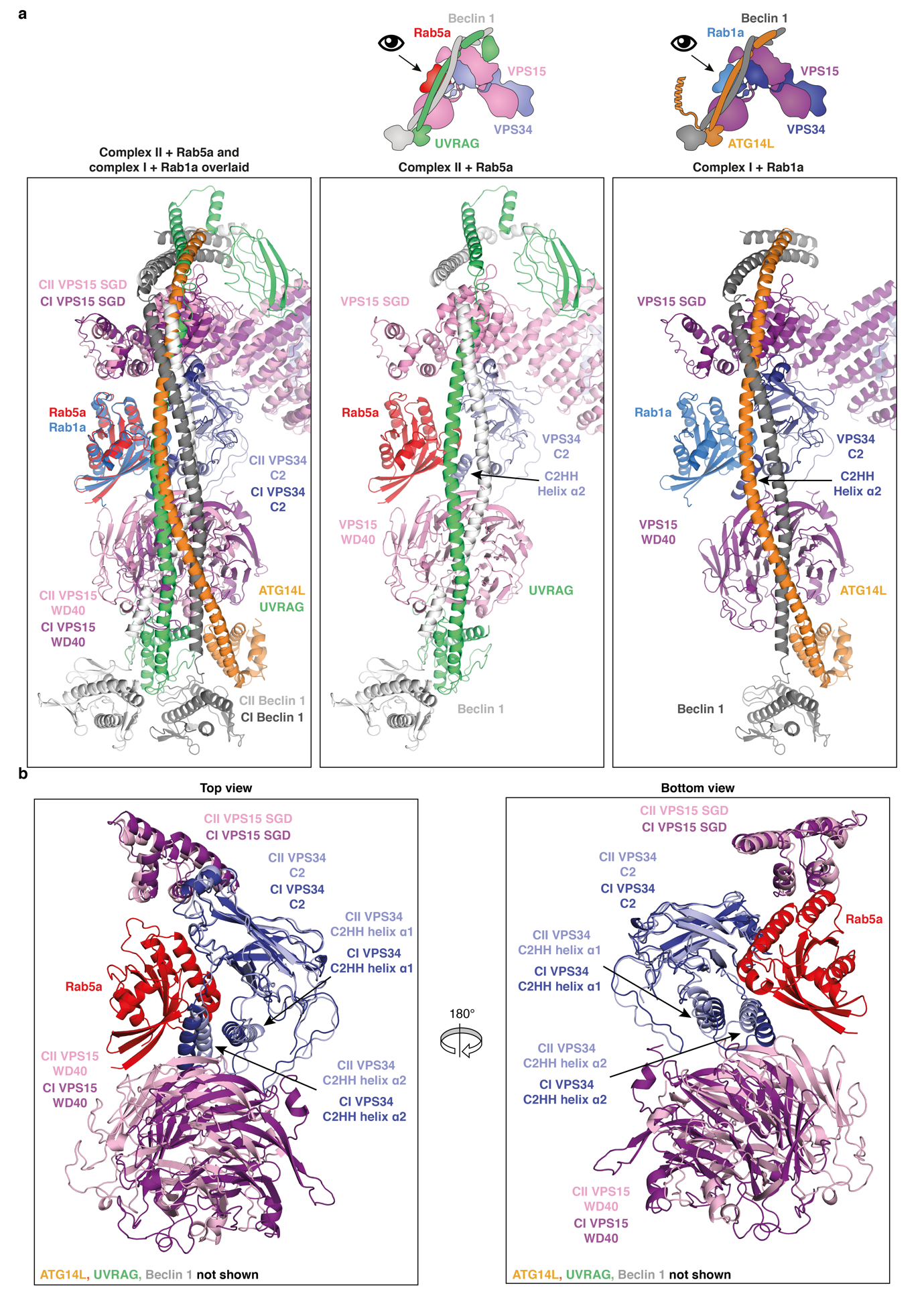
**

**Supplementary Figure 7 | Changes in conformation of the adaptor arm for complexes I and II.**

**a,** A view of the adaptor arm of complex I with Rab1a (right), complex II with Rab5a (middle) and an overlay of the two complexes (left). **b,** A closer view of the Rab-binding site formed from the VPS15 SGD, VPS15 WD40 and VPS34 C2 domain helical insertion (C2HH).


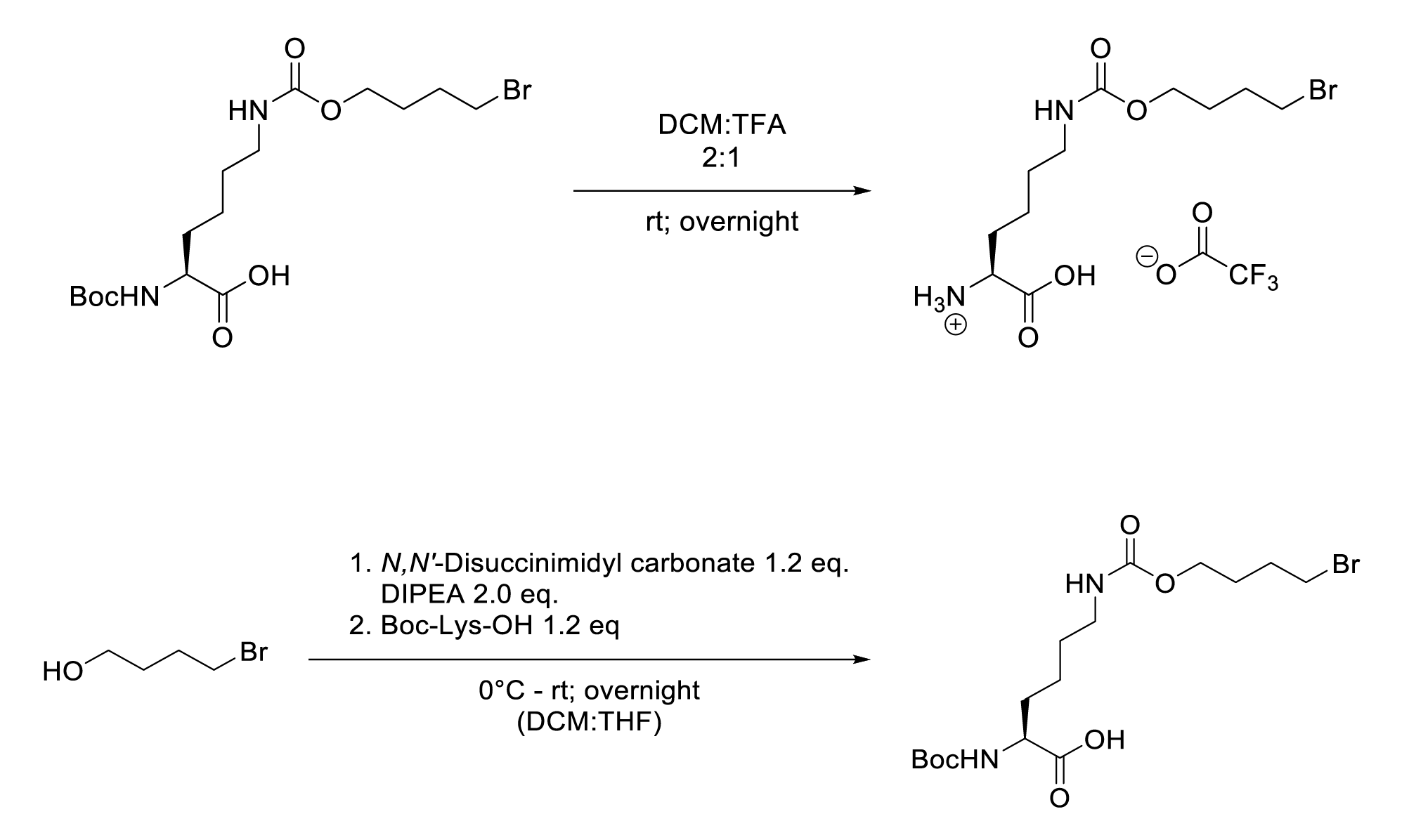


*α*-BocBrCO6K


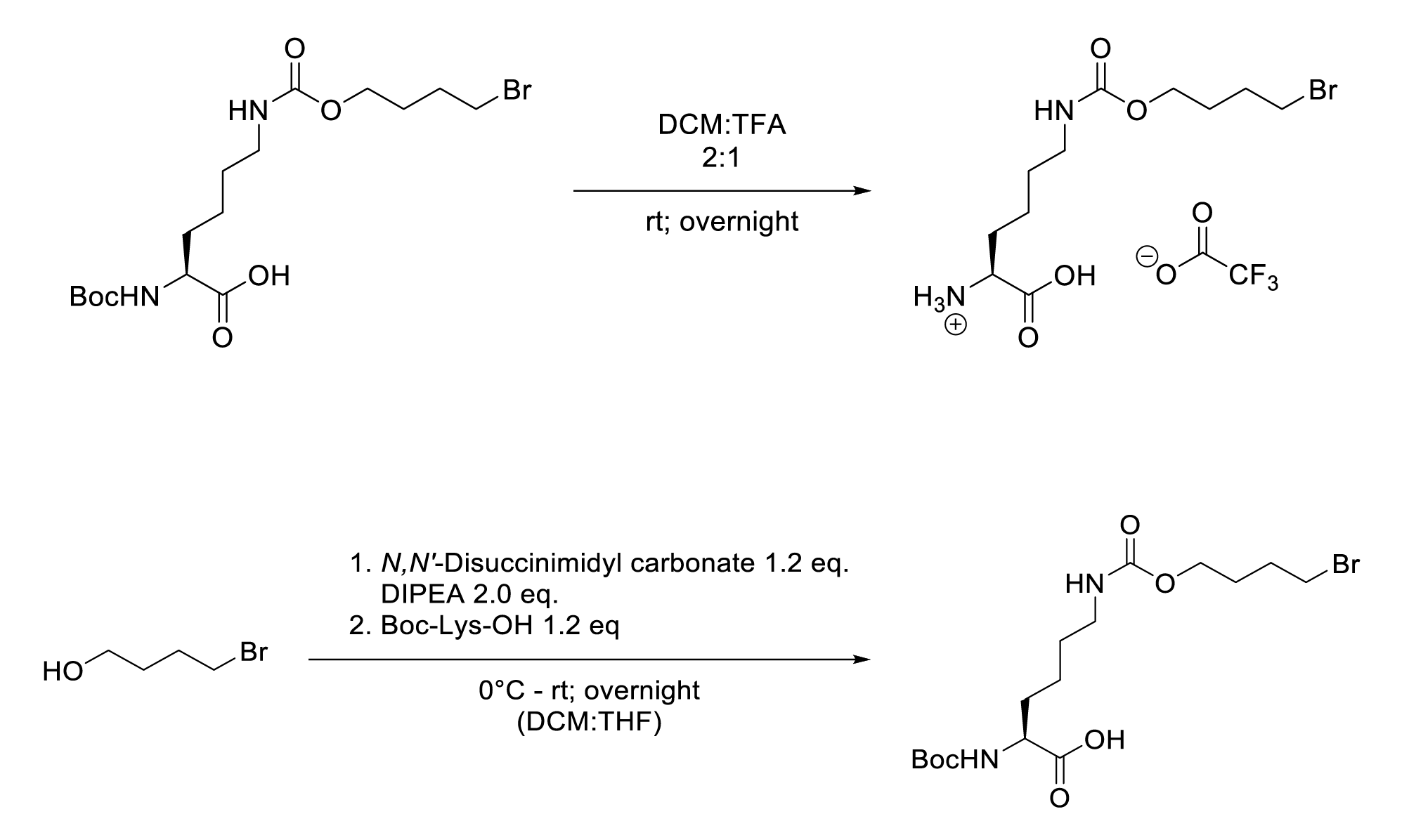


BrCO6K

**Supplementary Figure 8 | Synthesis of Synthesis of BrCO6K.**

The reaction scheme illustrates the synthesis of the unnatural amino acid BrCO6K that was incorporated into Rab5a for site-specific crosslinking of Rab5a with VPS34 complex II.


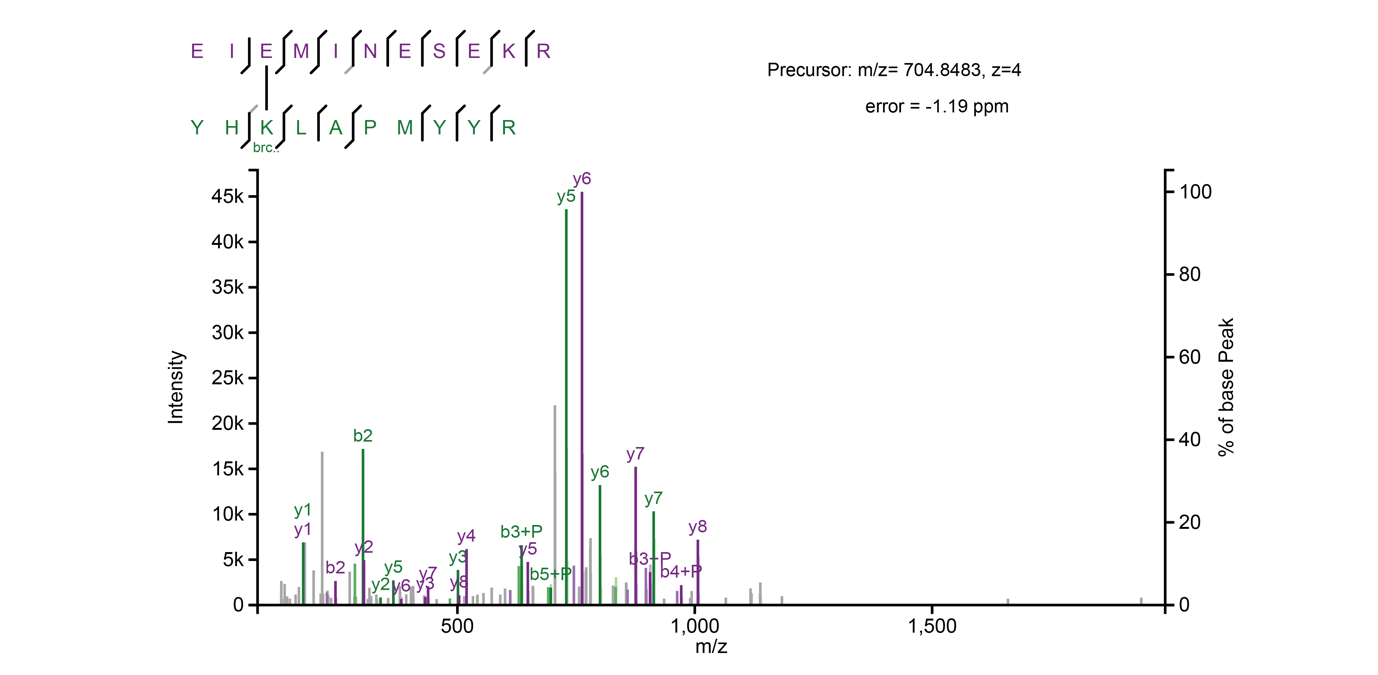


**Supplementary Figure 9 | Annotated fragmentation spectrum of crosslinked Rab5a-VPS34 peptide.**

The annotated fragmentation spectrum of crosslinked peptide EIE(xl)MINESEKR-YHKbrco6k(xl)LAPMYYR, which supports the identification of a crosslink between Rab5a residue 84BrCO6K and VSP34 residue E202.

**
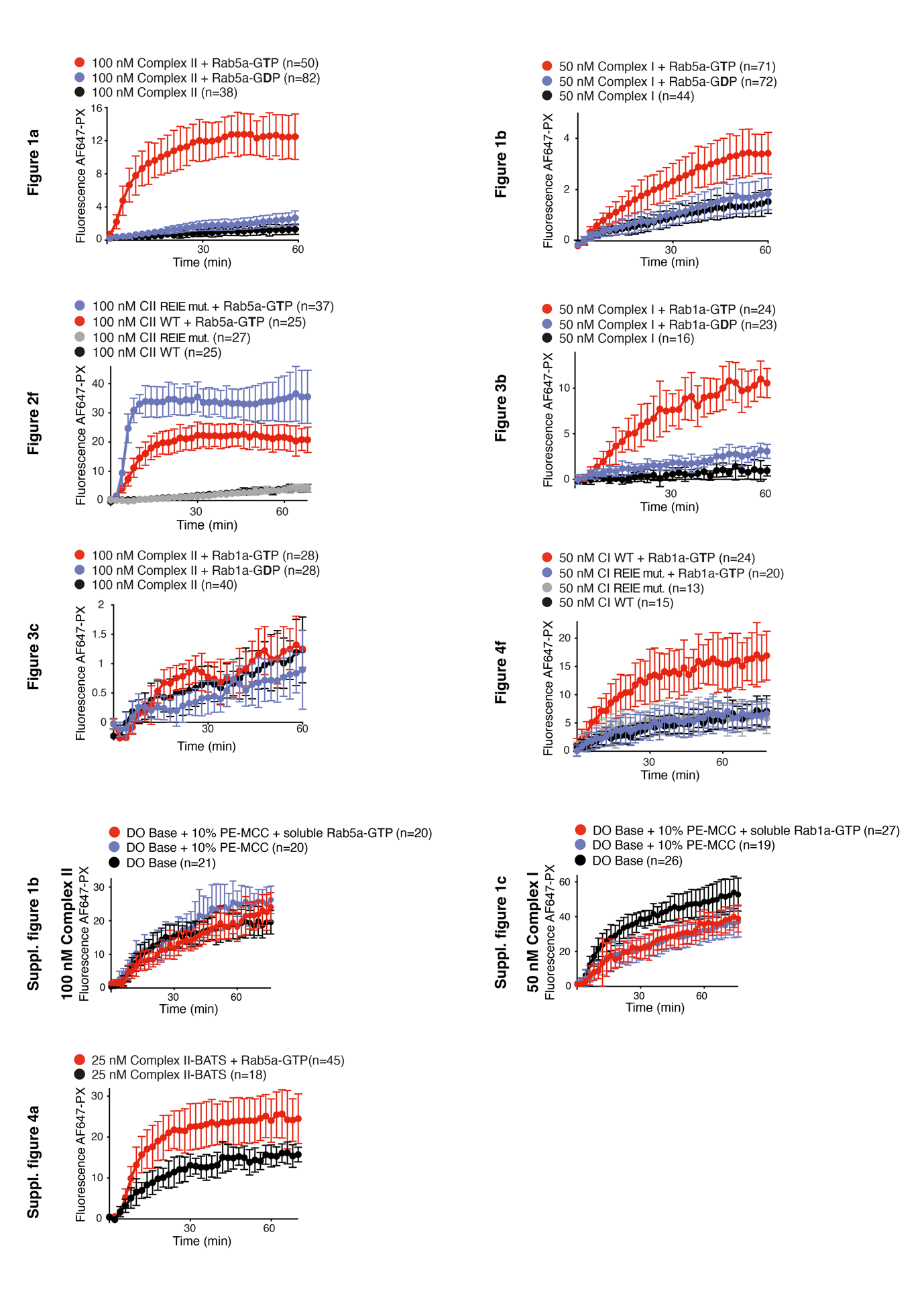
**

**Supplementary Figure 10 | Reaction time courses with error bars.**

Illustrations of the reaction progress curves with error bars for each time point calculated from the indicated number of GUVs (n).

**Supplementary Table 1 - UAA crosslinking table**

**Sample: Rab5a_human_Q79L_1-212_C19S_C63S_S84TAG and complex II (VPS34/VPS15/Beclin 1/UVRAG)**

| **Protein1** | **Linked**  **Residue1** | **PepSeq1** | **Link**  **Position**  **in Peptide1** | **Protein2** | **Linked**  **Residue2** | **PepSeq2** | **Link**  **Position**  **In**  **Peptide1** | **Number**  **Of**  **CSM** | **Highest**  **CSM**  **Score** | **Highest**  **Score**  **CSM**  **Mass**  **Error (ppm)** |
| --- | --- | --- | --- | --- | --- | --- | --- | --- | --- | --- |
| sp\|PK3C3  HUMAN | 202 | EIEMINESEKR | 3 | sp\|Rab5a  HUMAN | 84 | YHKbrco6kLAPMYYR | 3 | 3 | 14.42 | -1.194035455 |
| sp\|Rab5a  HUMAN | 84 | YHKbrco6kLAPMoxYYR | 3 | sp\|PK3C3  HUMAN | 202 | EIEMINESEKR | 3 | 5 | 14.4 | -1.586877845 |
| sp\|PK3C3  HUMAN | 202 | EIEMoxINESEKR | 3 | sp\|Rab5a  HUMAN | 84 | YHKbrco6kLAPMoxYYR | 3 | 1 | 12.29 | 0.328802003 |
| sp\|Rab5a  HUMAN | 84 | YHKbrco6kLAPMYYR | 3 | sp\|PK3C3  HUMAN | 202 | EIEMoxINESEKR | 3 | 3 | 12.05 | -0.086172111 |
| sp\|Rab5a  HUMAN | 84 | YHKbrco6kLAPMoxYYR | 3 | sp\|PK3C3  HUMAN | 202 | EIEMoxINESEK | 3 | 2 | 10.95 | 0.140392362 |
| sp\|Rab5a  HUMAN | 84 | YHKbrco6kLAPMYYR | 3 | sp\|PK3C3  HUMAN\| | 202 | EIEMINESEK | 3 | 1 | 10.41 | 0.038496226 |
| sp\|PK3C3  HUMAN | 202 | EIEMINESEK | 3 | sp\|Rab5a  HUMAN | 84 | YHKbrco6kLAPMoxYYR | 3 | 1 | 8.61 | 0.935143558 |

**Sample: Rab5a_human_Q79L_1-212_C19S_C63S_S84TAG and VPS34 alone**

| sp\|Rab5a  HUMAN | 84 | YHKbrco6kLAPMYYR | 3 | sp\|PK3C3  HUMAN | 202 | EIEMINESEKR | 3 | 1 | 8.69 | -0.507198269 |
| --- | --- | --- | --- | --- | --- | --- | --- | --- | --- | --- |

UAA crosslinking of Rab5a-S84BrCO6K to human complex II or VPS34 alone. A list of identified crosslinked peptide pairs is reported.

**Supplementary Table 2 - Plasmid table**

| **ID** | **Construct** | **Backbone** | **Marker** | **Use** |
| --- | --- | --- | --- | --- |
| pYO1025 | HsVPS34 untagged and  HsVPS15-3xTEV-ZZ tag | pCAG | amp | Cryo-ET,  GUV assays,  UAA,  HDX-MS |
| pYO1031 | HsBeclin1 and  HsUVRAG, both untagged | pCAG | amp | Cryo-ET,  GUV assays,  UAA,  HDX-MS |
| pYO1006 | HsBECLIN1 untagged | pCAG | amp | Cryo-ET,  Cell biology |
| pYO1124 | HsUVRAGΔCter(1-464) fused to HsATG14L BATS(413-492), untagged | pCAG | amp | Cryo-ET |
| STp8 (pOP823) | HsRab5a-Q79L-1-212-(C19S,C63S) | pOPINS | kan | GUV assays |
| pJB78 | HsRab1a-Q70L-1-204-(C26S,C126S) | pOPTG | amp | GUV assays |
| pYO1125 | GST-TEV-Cys-p40PX(1-128) | pOPTG | amp | GUV assays |
| pYO1274 | HsVPS34-(199-REIE-202>AAAA)  FL, untagged | pcDNA4/TO | amp | GUV assays |
| pYO1015 | HsVPS15-3xTEV-ZZ | pCAG | amp | GUV assays |
| pYO1101 | HsBeclin1 and  HsATG14L, both untagged | pCAG | amp | GUV assays, HDX-MS |
| pYO1261 | His6-SUMO-TEV-HsRab5a-Q79L-(1-211) | pOPTH | amp | HDX-MS |
| pYO1262 | His6-TEV-Rab1a-Q70L-(1-203) | pET28 | kan | HDX-MS |
| pSM41 | His6-TEV-HsVPS34 | pOPTH | amp | UAA |
| STp6 | HsRab5a-Q79L-1-212-(C19S,C63S,S84TAG)-His6 | pBAD | amp | UAA |
| STp12 (KL12) | TEMPOH-I_PylT (aa-tRNA synthetase for BrCo6K UAA and tRNA) | pEVOL | cam | UAA |
| pYO1280 | HsVPS34-EGFP | EGFP-C1 | kan | Cell biology |
| pYO350 | HsVPS15 untagged | pcDNA4/TO | amp | Cell biology |
| pYO1300 | HsVPS34-(199-REIE-202>AAAA)-EGFP | EGFP-C1 | kan | Cell biology |
| pYO1005 | HsUVRAG untagged | pCAG | amp | Cell biology |
| pYO1100 | HsATG14L untagged | pCAG | amp | Cell biology |
| pYO1296 | mCherry-HsRab5a-Q79L-(2-215) (FL) | EGFP-C1 | kan | Cell biology |
| pJB180 | mCherry-HsRab1a-Q70L-(1-205) (FL) | pcDNA3.1 | amp | Cell biology |
| JB28 | HsRab5a(Q79L)-BirA*-HA-MAO | pcDNA3.1 | amp | MitoID |
| JB40 | HsRab5a(S34N)-BirA*-HA-MAO | pcDNA3.1 | amp | MitoID |
| JB49 | HsRab1a(Q70L)-BirA*-HA-MAO | pcDNA3.1 | amp | MitoID |
| JB50 | HsRab1a(S25N)-BirA*-HA-MAO | pcDNA3.1 | amp | MitoID |
| JB74 | HsRab1a(WT)-BirA*-HA-MAO | pcDNA3.1 | amp | MitoID |

List of plasmids used in this study.

**Supplementary Table 3 - Lipid mixture table**

| **GUVs** | | | |
| --- | --- | --- | --- |
| **ID** | **Composition** | **Description in figure** | **Figure number** |
| STGUV11 | 18% liver PI, 10% DOPS,  7% DOPE, 55% DOPC,  10% DOPE-MCC,  0.03% DO Liss Rhod PE,  0.03% DSPE-PEG(2000) Biotin | DO Base + 10% PE-MCC | Fig. 1a,b  Fig. 2f,g  Fig. 3b,c  Fig. 4f,g  Suppl. Fig. 1  Suppl. Fig. 4a |
| YOGUV3 /  STGUV2 | 18% liver PI, 10% DOPS,  17% DOPE, 55% DOPC,  0.017% DO Liss Rhod PE  0.03% DSPE-PEG(2000) Biotin | DO Base | Suppl. Fig. 1b,c |
|  | | | |
| **LUVs** | | | |
| **ID** | **Composition** | **Used in** | **Figure number** |
| STSUV19 | 16% liver PI, 10% Brain PS,  12% Brain PE, 56% Brain PC,  5% DOPE-MCC,  0.1% DO Liss Rhod PE | Flotation +/- Rab GTPases | Fig. 1c  Fig. 3d  Suppl. Fig. 2 |
| STSUV32 | 16% liver PI,  12% DOPE, 66% DOPC,  6% DOPE-MCC,  0.2% DO Liss Rhod PE | Cryo-ET | Fig. 5a-c, e  Fig. 6a-d |
|  | | | |
| **Lipids** | | | |
| **Catalogue number** | **Product** | **Company** | |
| 850375C | DOPC | Avanti Polar Lipids, Inc | |
| 850725C | DOPE | Avanti Polar Lipids, Inc | |
| 840035C | DOPS | Avanti Polar Lipids, Inc | |
| 840053C | Brain PC (Porcine) | Avanti Polar Lipids, Inc | |
| 840022C | Brain PE (Porcine) | Avanti Polar Lipids, Inc | |
| 840032C | Brain PS (Porcine) | Avanti Polar Lipids, Inc | |
| 840042C | Liver PI (mixed chain PI, Bovine) | Avanti Polar Lipids, Inc | |
| 880129C | DSPE-PEG(2000) Biotin | Avanti Polar Lipids, Inc | |
| 810150C | DO Liss Rhod PE | Avanti Polar Lipids, Inc | |
| 780201C | DOPE-MCC | Avanti Polar Lipids, Inc | |

GUV/LUV lipid mixtures and lipids used in this study.

**Supplementary Table 4 – Cryo-ET data acquisition and image processing**

| **EMDB entries** | EMD-12214, EMD- EMD-12237, EMD-12238 |
| --- | --- |
| Voltage (kV) | 300 |
| Detector | Gatan K3 |
| Energy filter slit width (eV) | 20 |
| Electron exposure  (e^-^/ Å^2^) | ~ 120 |
| Defocus range (µm) | -2.5 to -5 |
| Tilt range (min/max, step) | -60°/+60, 3° |
| Tilt scheme | dose-symmetrical (Hagen scheme) |
| Tomogram used/acquired (no.) | 105/115 |
| Pixel size (Å) | 2.133 |
| Vesicles (no.) | 2,896 |
| Symmetry imposed | none |
| Final subtomograms (no.) | 26,979 |
| Map resolution (Å) | 9.8 |
| FSC threshold | 0.143 |
| Map resolution range (Å) | 8-16 |

Parameters of the cryo-ET data collection and processing workflow.
