# Supplementary material for: Structural basis for VPS34 kinase activation by Rab1 and Rab5 on membranes
Source: Nat Commun. 2021 Mar 10;12:1564. doi: 10.1038/s41467-021-21695-2 (PMC7946940; doi:10.1038/s41467-021-21695-2)
Supplement: Supplementary file 7 — Description of Additional Supplementary Files [file 41467_2021_21695_MOESM7_ESM.docx]

Description of Additional Supplementary information

Title: Supplementary Movie 1.

Description: Fit of the density to Rab5a. A view of the Rab5a bunding site, rotating around the x-axis to illustrate the unambiguous fit of the model to the density.

Title: Supplementary Movie 2.

Description: The VPS34 complex II can adopt a range of orientations with respect to the membrane. The rhree classes shown in Fig. 6d were arranged in sequence by increasing angle with respect to the membrane and shown in two orthogonal views.

Title: Supplementary Data 1.

Description: HDX-MS observed for human VPS34 complex II peptides in the presence and absence human Rab5a. Every peptide included within the dataset is shown, along with the quality assessment statistics of the dataset. Each subunit of the complex is a separate Excel worksheet.

Title: Supplementary Data 2.

Description: HDX-MS observed for human VPS34 complex I peptides in the presence and absence human Rab1a. Every peptide included within the dataset is shown, along with the quality assessment statistics of the dataset. Each subunit of the complex is a separate Excel worksheet.
