# Supplementary material for: Structural basis for VPS34 kinase activation by Rab1 and Rab5 on membranes
Source: Nat Commun. 2021 Mar 10;12:1564. doi: 10.1038/s41467-021-21695-2 (PMC7946940; doi:10.1038/s41467-021-21695-2)
Supplement: Supplementary file 8 — Reporting Summary [file 41467_2021_21695_MOESM8_ESM.pdf]

nature research

Corresponding author(s): Roger L Williams
John A G Briggs
Sean Munro

Last updated by author(s): Jan 21, 2021

Reporting Summary

Nature Research wishes to improve the reproducibility of the work that we publish. This form provides structure for consistency and transparency in reporting. For further information on Nature Research policies, see our Editorial Policies and the Editorial Policy Checklist.

Statistics

For all statistical analyses, confirm that the following items are present in the figure legend, table legend, main text, or Methods section.

n/a	Confirmed	
☐	☒	The exact sample size (*n*) for each experimental group/condition, given as a discrete number and unit of measurement
☐	☒	A statement on whether measurements were taken from distinct samples or whether the same sample was measured repeatedly
☐	☒	The statistical test(s) used AND whether they are one- or two-sided<br>*Only common tests should be described solely by name; describe more complex techniques in the Methods section.*
☒	☐	A description of all covariates tested
☒	☐	A description of any assumptions or corrections, such as tests of normality and adjustment for multiple comparisons
☐	☒	A full description of the statistical parameters including central tendency (e.g. means) or other basic estimates (e.g. regression coefficient) AND variation (e.g. standard deviation) or associated estimates of uncertainty (e.g. confidence intervals)
☐	☒	For null hypothesis testing, the test statistic (e.g. F, t, r) with confidence intervals, effect sizes, degrees of freedom and P value noted<br>*Give P values as exact values whenever suitable.*
☒	☐	For Bayesian analysis, information on the choice of priors and Markov chain Monte Carlo settings
☒	☐	For hierarchical and complex designs, identification of the appropriate level for tests and full reporting of outcomes
☒	☐	Estimates of effect sizes (e.g. Cohen's *d*, Pearson's *r*), indicating how they were calculated

Our web collection on statistics for biologists contains articles on many of the points above.

Software and code

Policy information about availability of computer code

Data collection	Serial EM 3.8.0 for operation of electron microscope (available and referenced in the Methods section)<br>Confocal images were collected with ZEN 2.1 SP3
Data analysis	CryoET data processing (all commercial or available and referenced in methods): IMOD 4.10; NovaCTF 1.0.0, EMAN2.2; Matlab (Version R2016b); TOM (no version number); AV3 (no version number); subTOM (no version number, available for download from https://www2.mrc-lmb.cam.ac.uk/groups/briggs/resources] and [https://github.com/DustinMorado/subTOM/releases/tag/v1.1.4); Python 3.7; RELION 3.1.<br>HDX data were analysed by DynamX HDX Data Analysis Software 3.0 : Waters<br>Peptide identification for HDX Protein Lynx Global Server (Version 3.0.1, PLGS, Waters, U.K.).<br>Modelling/structure refinement/visualization (all available and referenced in methods): Coot 0.9; REFMAC 5.8.0272; Chimera 1.14; PyMOL 2.4.0, ISOLDE 1.1, ChimeraX-1.1.1, REFMAC<br>GUV intensities were analyzed by ImagaJ (Version 2.0.0-rc-69/1.52i), with a custom macro as described in the text and Adobe Photoshop (Version 2015.5.0)<br>GUV kinetics were analyzed with Prism 7 (GraphPad Software)<br>Cross-links were analyzed with MSConvert in ProteoWizard (version 3.0.11729) and xiSEARCH software (version 1.7.6 https://www.rappsilberlab.org/<br>software/xisearch)

For manuscripts utilizing custom algorithms or software that are central to the research but not yet described in published literature, software must be made available to editors and reviewers. We strongly encourage code deposition in a community repository (e.g. GitHub). See the Nature Research guidelines for submitting code & software for further information.

Data

Policy information about availability of data

All manuscripts must include a data availability statement. This statement should provide the following information, where applicable:

- Accession codes, unique identifiers, or web links for publicly available datasets
- A list of figures that have associated raw data
- A description of any restrictions on data availability

The cryo-ET reconstruction has been deposited to EMDB (EMD-12214) and the coordinates of a model to PDB (Accession code 7BL1). For initial models, PDB entries 5DFZ (yeast complex II), 3IHY (human VPS34 HELCAT), 3MJH (human Rab5a/GTP) and 4DDP (human Beclin1 BARA domain)
CLMS data were deposited to ProteomeXchange (aka PRIDE) (accession code PXD023533) and jPOST (accession code JPST001056).

Field-specific reporting

Please select the one below that is the best fit for your research. If you are not sure, read the appropriate sections before making your selection.

☒ Life sciences ☐ Behavioural & social sciences ☐ Ecological, evolutionary & environmental sciences

For a reference copy of the document with all sections, see nature.com/documents/nr-reporting-summary-flat.pdf

Life sciences study design

All studies must disclose on these points even when the disclosure is negative.

Sample size	For the tomography, we collected all data that were possible within the time on the microscope that we had. The sample size is sufficient to obtain a structure at the reported resolution, as assessed by Fourier shell correlation. For HDX and kinetic assays, we made sufficient independent measurements to estimate the error of the measurements. For GUV assays, the sample size corresponds to the number of vesicles that were analyzed and is listed on each figure with GUV results.
Data exclusions	For all cryo-ET experiments, tomograms that could not be used due to objects (contamination, ice, grid bars etc) obscuring the vesicle were discarded. Exclusion of error-containing or misaligned data is standard for cryo-ET data processing. Misaligned sub-tomograms were excluded from averages based on cross-correlation scores and minimal distance threshold, as described in the methods.<br><br>For HDX, peptide inclusion criteria was a minimum intensity of 5000, a minimum sequence length of 5 amino acids, a minimum of 0.1 products per amino acid, a maximum MH+ error of 5 ppm, and a positive identification meeting these criteria in at least 2 of the three non-deuterated files. An initial automated spectral processing step was conducted by DynamX followed by a manual inspection of individual peptides for sufficient quality.<br><br>Cross-link peptide inclusion was based on MS accuracy = 4 ppm; MS2 accuracy = 10 ppm; enzyme = trypsin (with full tryptic specificity); allowed number of missed cleavages = 4; missing monoisotopic peak=2; fixed modifications = carbamidomethylation on cysteine; variable modifications = oxidation on methionine
Replication	For CryoET, division of dataset into two random halves was done based on particle number.
Randomization	Blinding was not applicable to this study because this type of study does not use group allocation.
Blinding	Blinding was not applicable to this study because this type of study does not use group allocation.

Reporting for specific materials, systems and methods

We require information from authors about some types of materials, experimental systems and methods used in many studies. Here, indicate whether each material, system or method listed is relevant to your study. If you are not sure if a list item applies to your research, read the appropriate section before selecting a response.

Materials & experimental systems

n/a	Involved in the study	
☐	☒	Antibodies
☐	☒	Eukaryotic cell lines
☒	☐	Palaeontology and archaeology
☒	☐	Animals and other organisms
☒	☐	Human research participants
☒	☐	Clinical data
☒	☐	Dual use research of concern

Methods

n/a	Involved in the study	
☒	☐	ChIP-seq
☒	☐	Flow cytometry
☒	☐	MRI-based neuroimaging

Antibodies

Antibodies used	Cell Signaling Technology:<br>1. Anti-Rab5 Antibody (Cell Signaling Technology, 2143S, lot number 3)<br>2. Rabbit anti-ATG14L (Cell Signaling Technology, 5504S, lot number 2)<br>3. Rabbit anti-UVRAG (Cell Signaling Technology, 5320S, lot number 1)<br><br>Thermo Fisher<br>4. Goat anti-rabbit secondary antibody Alexa Fluor 488 (Thermo Fisher, A-11008)<br><br>Proteintech<br>5. Rabbit anti-VPS15 (Proteintech, 17894-1-AP, lot number unknown)<br>6. Rabbit anti-VPS34 (Proteintech, 12452-1-AP, lot number 00058079)<br><br>Santa Cruz<br>7. Rabbit anti-Beclin1 (Santa Cruz Biotechnology, sc-11427, lot #H3012)<br><br>Produced ourselves<br>8. Anti-HA antibody was the supernatant from a hybridoma clone 12CA5, antibody registry AB_2532070
Validation	1. Anti-Rab5 Antibody (Cell Signaling Technology, 2143S)<br>Validation statement from the vendor's website:<br>Rab5A Antibody detects endogenous levels of total Rab5A protein.<br>Species Reactivity: Human, Mouse, Rat, Hamster, Monkey<br><br>2. Rabbit anti-ATG14L (Cell Signaling Technology, 5504S)<br>Validation statement from the vendor's website:<br>Atg14 Antibody detects endogenous levels of total Atg14 protein.<br>Species Reactivity: Human, predicted to react based on 100% sequence homology: Monkey<br><br>3. Rabbit anti-UVRAG (Cell Signaling Technology, 5320S)<br>Validation statement from the vendor's website:<br>UVRAG Antibody detects endogenous levels of total UVRAG protein. Species Reactivity: Human, Mouse<br><br>5. Rabbit anti-VPS15 (Proteintech, 17894-1-AP)<br>Validation statement from the vendor's website:<br>17894-1-AP targets VPS15 in WB, IF, ELISA applications and shows reactivity with human, mouse, rat samples.<br><br>6. Rabbit anti-VPS34 (Proteintech, 12452-1-AP)<br>Validation statement from the vendor's website:<br>12452-1-AP targets VPS34 (C terminal) in WB, IHC, IF, ELISA applications and shows reactivity with human, mouse, rat samples.<br><br>7. Rabbit anti-Beclin1 (Santa Cruz Biotechnology, sc-11427, lot #H3012)<br>Validation statement from the vendor's website:<br>BECN1 (H-300) is recommended for detection of BECN1 of mouse, rat and human origin by Western Blotting (starting dilution 1:200, dilution range 1:100-1:1000), immunoprecipitation [1-2 µg per 100-500 µg of total protein (1 ml of cell lysate)], immunofluorescence (starting dilution 1:50, dilution range 1:50-1:500) and immunohistochemistry (including paraffin-embedded sections) (starting dilution 1:50, dilution range 1:50-1:500).<br>BECN1 (H-300) is also recommended for detection of BECN1 in additional species, including equine, canine, bovine, porcine and avian.<br><br>8. Anti-HA antibody was the supernatant from a hybridoma clone 12CA5<br>Validation statement from antibody registry:<br>raised against influenza hemagglutinin peptide HAl (residues 75-110)

Eukaryotic cell lines

Policy information about cell lines

Cell line source(s)	HEK293T (ATCC CRL-3216), Expi293 suspension cells (ThermoFisher A14527)
Authentication	All cell lines were used without authentication
Mycoplasma contamination	The cell lines for protein production were not routinely tested for mycoplasma contamination. The HEK293T cells used for microscopy were tested for mycoplasma.
Commonly misidentified lines<br>(See ICLAC register)	None
